# A Second Trimester Abdominal Ectopic Pregnancy Visualised Using Standard and Handheld Point‐of‐Care Ultrasound: A Case Report

**DOI:** 10.1002/ajum.70013

**Published:** 2025-07-09

**Authors:** Nina Cooper, Alex Novak, Georgina Gould, Shaun Haran, Maeve Tuomey, Maya Al‐Memar, Catriona Stalder, Tom Bourne, Joseph Yazbek

**Affiliations:** ^1^ Queen Charlotte's and Chelsea Hospital Imperial College Healthcare NHS Trust London UK; ^2^ Department of Metabolism, Digestion and Reproduction Imperial College London London UK; ^3^ Department of Surgery and Cancer Imperial College London London UK

**Keywords:** acute gynaecology, early pregnancy, ectopic pregnancy, medical imaging, POCUS, sonography

## Abstract

**Background:**

A 27‐year‐old nulliparous woman with a history of subfertility presented to a routine dating scan at 13 + 1 weeks gestation with a history of left‐sided abdominal pain.

**Key Findings:**

Both standard transabdominal ultrasound and handheld point‐of‐care ultrasound (POCUS) identified a probable abdominal ectopic pregnancy (AEP), confirmed by MRI. Intraoperative findings revealed a live pregnancy encapsulated by the omentum with associated haemoperitoneum. Active haemorrhage necessitated conversion from laparoscopy to laparotomy, culminating in a left salpingo‐oophorectomy.

**Discussion:**

This case highlights the potential role of handheld POCUS in diagnosing a rare and life‐threatening condition. The device enabled immediate, bedside imaging and facilitated rapid decision‐making. To our knowledge, this is the first documented case of an abdominal ectopic pregnancy diagnosed using handheld POCUS, emphasising its potential to improve maternal outcomes through early detection and intervention.

## 1 Introduction

Abdominal ectopic pregnancy (AEP) describes implantation of a pregnancy within the abdominal cavity, which may be primary (implantation directly outside of the abdomen) or secondary (implantation after tubal rupture) [1]. Due to its rarity, the exact incidence is uncertain but is estimated at 1%–2% of all EPs [2].

Early AEP is classified as those identified prior to 20 weeks gestation. The mean gestation at diagnosis is typically 10 + 0 weeks, most are found in the uterovesical or rectouterine pouches or overlying the uterine serosa [3]. Most cases (87.9%) are managed surgically and are associated with major haemorrhage [3]. The mortality rate is 7.7 times higher than that of tubal ectopic pregnancy (EP) [4]. Prompt identification can mean the difference between life and death, especially where access to imaging is limited. Handheld point of care ultrasound (POCUS) may offer an immediate diagnosis; however, the use of POCUS in AEP has not yet been explored.

## 2 Case Presentation

We describe a 27‐year‐old woman presenting with abdominal pain at 13 + 1 weeks gestation. She had a history of low ovarian reserve, subfertility, and a multi‐fibroid uterus (57 × 47 mm fundal subserosal, 38 × 33 mm posterior intramural, 31 × 25 mm posterior subserosal, 28 × 28 mm anterior subserosal, plus multiple smaller fibroids measuring up to 16 mm in size, uterine volume 306 cc). She spontaneously conceived after 3 years of trying. She had no known imaging features of endometriosis. She had a normal hormone profile but a low anti‐Müllerian hormone (AMH) of 2.4 pmol/L. She attended for a routine dating scan with a history of left lower quadrant pain. On examination, her abdomen was soft and diffusely tender in the suprapubic region.

### 2.1 Ethics Statement

Informed, written consent was obtained from the patient for publication of this case report. No ethical approval was required.

### 2.2 Imaging and Investigations

**FIGURE 1:**
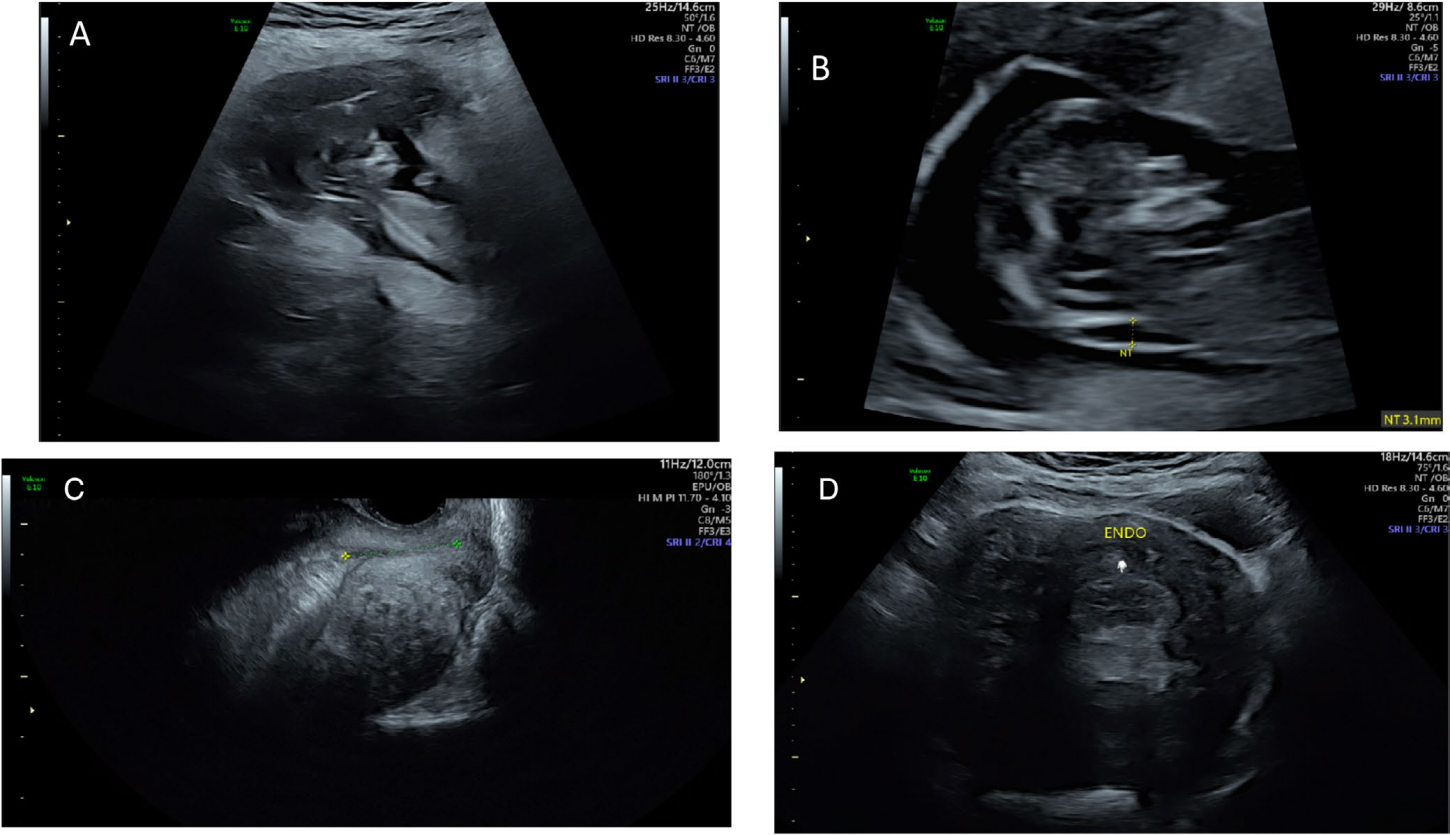
Ultrasound images. (A, B) Initial departmental ultrasound images demonstrating a fetus with a crown‐rump length (CRL) of 69 mm and nuchal translucency of 3.1 mm. The fetal anatomy and amniotic fluid volume was described as normal. The placenta was described as posterior. (C, D) Second ultrasound within early pregnancy unit scan demonstrating empty uterine cavity both transvaginally and transabdominally.

**FIGURE 2:**
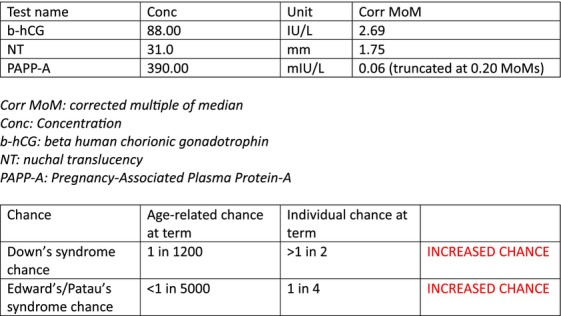
Combined screening test results.

The initial dating TAUS (Figure 1A,B) was reported as a probable intrauterine pregnancy (IUP). A combined screening test (CST) gave a 1 in 2 chance for trisomy 21 and a 1 in 4 chance for trisomies 13 and 18 (Figure 2).

**FIGURE 3:**
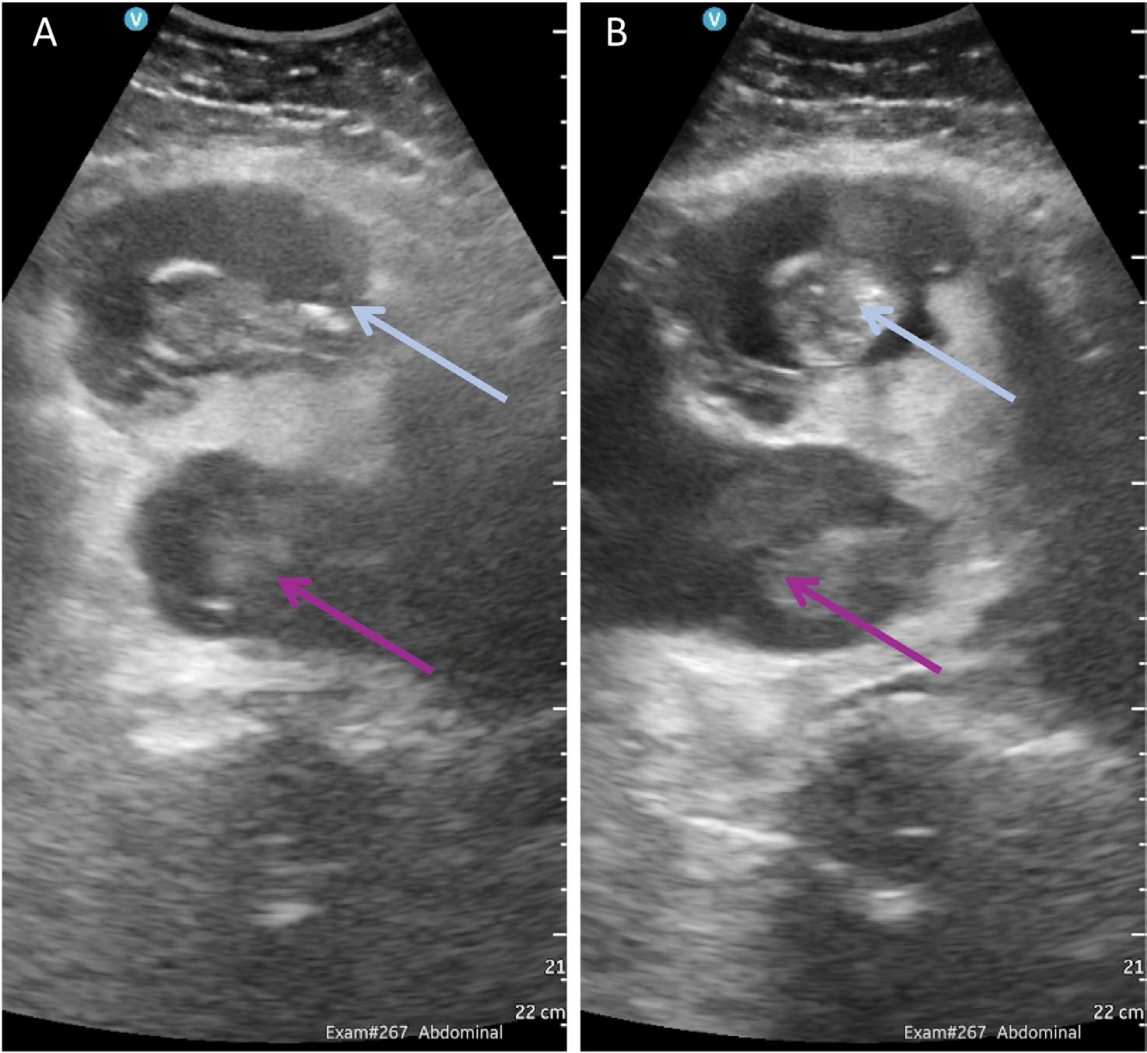
Point of care ultrasound images. Sagittal (A) and transverse (B) images of uterus (purple) and gestation sac (blue) on point of care ultrasound.

**FIGURE 4:**
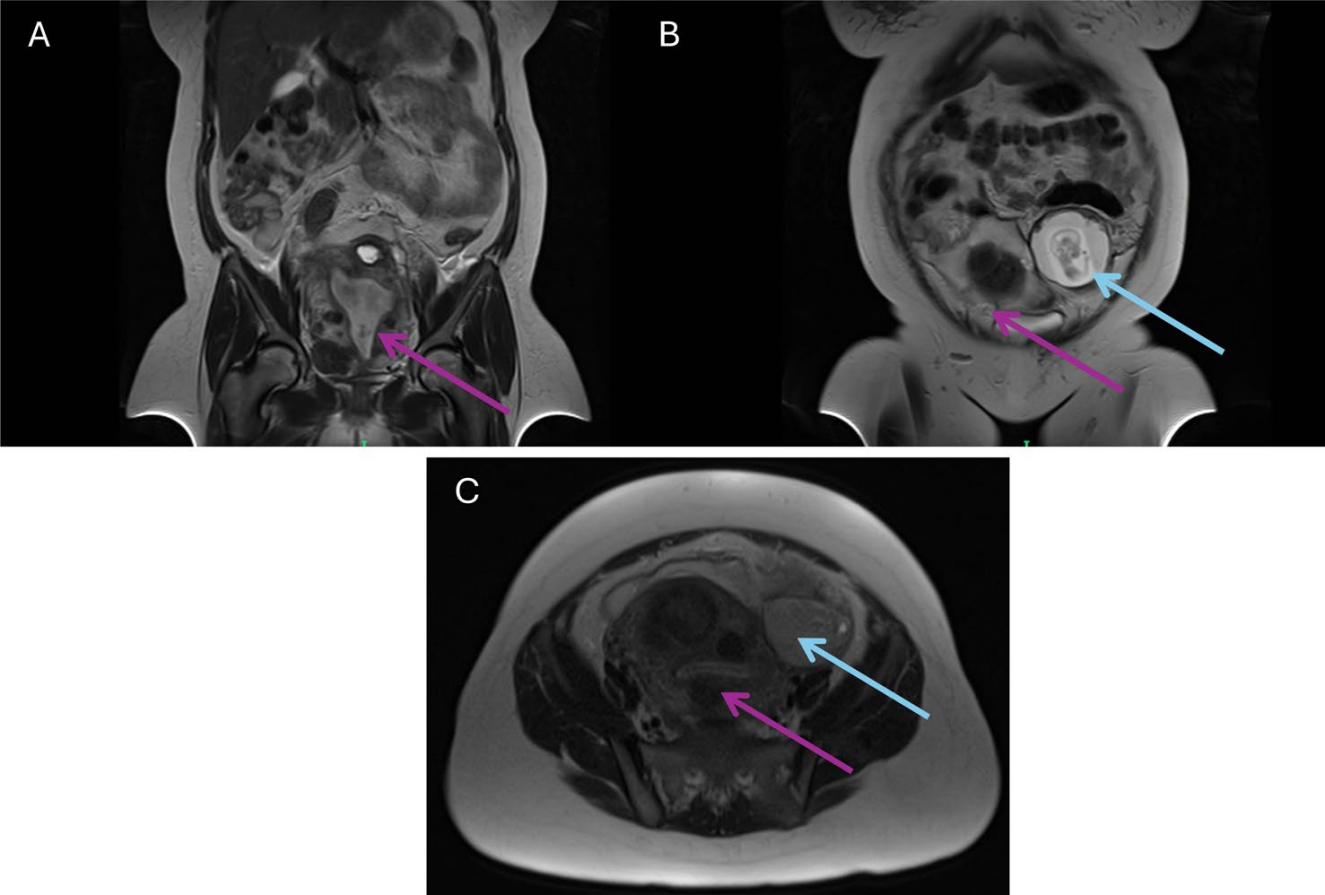
MRI images. (A, B) Coronal view of multi‐fibroid uterus (pink arrow) and pregnancy sac (blue arrow). (C) Axial view of uterus with left sided gestation sac visible. MRI showing the left adnexal pregnancy. The left ovary was seen separately. There was peripheral haemorrhage surrounding the gestation sac with a moderate intraperitoneal haematoma and free fluid within the perihepatic space and paracolic gutters.

Repeat TA and TV US by a level 3 nurse sonographer (GE Voluson E10) (Figure 2C,D) demonstrated a multi‐fibroid uterus and an extrauterine pregnancy with an empty uterine cavity and visible ‘endometrial stripe’. The operating surgeon (EFSUMB level 3) used a handheld ultrasound (GE Voluson Air) to facilitate pre‐operative counselling in the ward setting. This allowed for contextualisation of the rationale for surgery and planned surgical approach alongside ‘real time’ images displayed on a mobile phone. The handheld images (Figure 3) demonstrated an empty uterine cavity and an extrauterine pregnancy in the left adnexa with no visible continuity with the cervix. The relationship to the ipsilateral ovary could not be assessed. Same‐day MRI showed a left adnexal EP which was presumed to be within the fallopian tube (Figure 4). Blood test results are summarised in Table 1.

**TABLE 1:** Blood results.

Blood test	Result
Haemoglobin	92 g/L
White blood cell count	9.7 × 10^9^/L
Platelet count	284 × 10^9^/L
Sodium	139 mmol/L
Potassium	3.5 mmol/L
Creatinine	41 umol/L
Urea	1.4 mmol/L
C‐reactive protein	133 mg/L
BhCG	119,656 IU/L

### 2.3 Therapeutic Intervention

The patient was informed of the findings and surgical management was recommended. A consultant‐led multi‐disciplinary team (MDT) including gynaecologists, haematologists, and radiologists managed this case. In our institution complex gynaecological surgery is supported by the gynae oncology service with access to general surgeons as required. Current UK practice does not warrant abortion consent to be completed for ectopic pregnancy, and as such this was not deemed legally required in this case.

**FIGURE 5:**
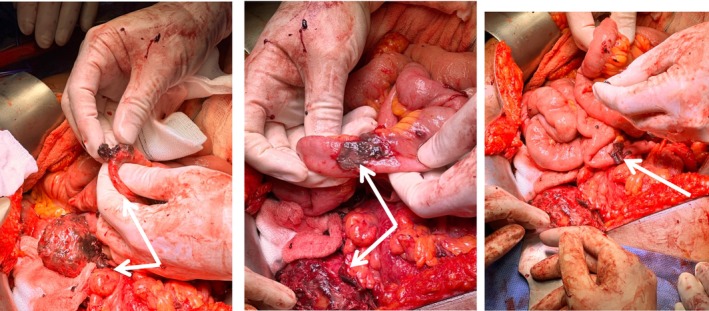
Intraoperative images showing trophoblast and haemosiderin deposits within the intraabdominal cavity, including involvement of bowel serosa (white arrows).

A diagnostic laparoscopy via Palmer's point entry was initially performed. Direct visualisation of the pregnancy noted it to be enveloped by overlying omentum. Extensive perihepatic and abdominal adhesions were noted. The omentum was dissected to reveal the gestational sac with a live fetus. Cessation of cord pulsation was performed with a handheld thermal device (Thunderbeat, Olympus Medical Systems). Due to ongoing active resuscitative measures, a decision to convert to laparotomy was made for life‐saving purposes. Active haemorrhage was noted from feeding neovascularised vasculature associated with the AEP. Both small and large bowel serosa had adherent trophoblastic tissue overlying (Figure 5). The fimbrial fallopian tube was grossly inflamed with haemosiderin and haemorrhagic clots adjacent. Despite attempts to salvage the ovary, the bleeding was such that an adnexectomy was performed due to ongoing transfusion requirements. Trophoblast was excised and a left salpingo‐oophorectomy was performed. There was no macroscopic evidence of endometriosis. The total blood loss was 2000 mL and the patient was admitted to the intensive care unit (ICU) for one night. Total blood products administered were three units of packed red blood cells and two units of fresh frozen plasma.

### 2.4 Outcome and Follow‐Up

The patient was discharged on day 9 post‐operatively, after recovery from post‐operative ileus. She completed outpatient follow‐up via early pregnancy unit with serial serum beta human chorionic gonadotrophin (BhCG) levels which declined to < 15 IU/L after 3 weeks. She was re‐referred to fertility services and for appropriate psychological support.

### 2.5 Histology

The histology confirmed a para‐ovarian abdominal ectopic pregnancy. There were decidualised cells within the omentum. The Fallopian tube had significant salpingitis and partially infarcted chorionic villi showing the location of initial implantation, with trophoblastic cells infiltrating the surrounding tissue, and possible features of prior endometriosis. The left ovary showed a corpus luteum, congestion, and granulation tissue on its surface.

### 2.6 Patient Perspective

“I never expected to lose my baby, that was a big thing for me. I had been trying for three years. I did not know what to do or what to decide. All the team has supported me and made me feel better… I have pain but I am getting through it.”

## 3 Discussion

Point of care ultrasound findings have been shown to accurately correlate with standard TVUS findings in early pregnancy and have a role as a screening tool to signpost to further imaging [5, 6]. This case demonstrates that an AEP can be classified as extrauterine using a transabdominal approach with a handheld ultrasound device. As far as the authors are aware, this is the first case to demonstrate an AEP with this modality. Comparing the image quality (Figure 1 vs. 3), handheld US provides lower resolution images but clearly demonstrates that the pregnancy is separate from the cavity. Similarly, handheld ultrasound has been shown to confidently identify pregnancy location in an unselected early pregnancy population (*k* = 0.785, substantial agreement) [5]. This supports the argument that handheld POCUS has the potential to provide life‐saving diagnostics in the emergency setting where a patient cannot be transferred to a scanning department, as well as in the low‐income setting where standard ultrasound may not be accessible [7].

There are cases in the literature of AEPs which were unrecognised as extrauterine due to the placenta implanting exclusively on the uterine serosa, in some instances being mistaken for an intrauterine pregnancy associated with a uterine anomaly. In some cases, pregnancies have progressed to a viable gestation or even term, and were subsequently identified as abdominal at delivery [8, 9]. Like other cases, where implantation is not exclusively uterine, this case demonstrated infiltration of trophoblast onto the surrounding bowel or omentum. The literature suggests such cases are at greater risk of complication [10].

Implementing ultrasound in the low resource setting is not just limited by the physical presence of machines, but also the training, maintenance, and infrastructure required to safely provide it. Currently, only 1/3 of women in low‐income settings have access to ultrasound [11, 12]. POCUS using smaller hand‐held devices has the potential to improve access; however, the technology still relies on a trained provider. Education is important to facilitate the safe roll‐out of these devices.

In the low resource setting, surgery may need to be life‐saving rather than organ‐saving. The unpredictable nature of these pregnancies warrants urgent consideration of a surgical approach with a low threshold to consider life‐saving methods for haemorrhage control [13]. A case series of 163 AEPs showed a maternal mortality of 12% and a perinatal mortality of 72% [14]. In low resource settings, AEP should be suspected where patients present with an acute abdomen, painful or absent fetal movements, or abdominal pain in the absence of a confirmed IUP, regardless of gestation. Handheld ultrasound is a useful immediate adjunct to physical examination without the need for transfer to an imaging department.

Although MRI confirmed an extrauterine pregnancy, the imaging report of a tubal ectopic pregnancy did not correlate with surgical findings. It is, however, possible that this was a secondary abdominal ectopic pregnancy which initially did implant within the tube. Implantation within the ovary was less likely given the histological findings of granulation tissue only on the ovary's surface. Confident mapping of the anatomical relationship of the EP to the abdominal viscera allows for early and prompt pre‐operative liaison with other specialities [15]. Two separate operators using standard and handheld ultrasound were able to identify an extrauterine pregnancy but could not identify the implantation site. Given its lateral position to the uterus, the EP was suspected to be tubal or ovarian. In cases where the patient is haemodynamically stable, point of care diagnostics may prompt transfer to a specialist unit to facilitate an organ‐sparing and/or fertility‐sparing approach. As a minimum, provision of adequate access to emergency blood products and critical care input should be available. We strongly recommend that all cases have pathways in place for the implementation of an emergency MDT.

In this specific case, the combined screening test showed an increased chance of all trisomy 13, 18, and 21 (Figure 2). Pregnancy associated plasma protein‐A (PAPP‐A) is a placenta‐derived glycoprotein produced by the trophoblast cells, which typically rises gradually in early pregnancy in a normally‐sited IUP. It was extremely low at 0.06 MoM. In the context of an AEP, a low PAPP‐A may support this as a potential differential diagnosis where cross‐sectional imaging is not readily available [16].

The psychological impact of an EP should not be underestimated. Using handheld ultrasound, we could counsel the patient in a ward setting with real‐time images. A prospective study of 186 women experiencing early pregnancy loss shows many women fulfil the diagnostic criteria for post‐traumatic stress disorder and moderate–severe anxiety [17]. In this case, the patient reported that the impact of the loss of a wanted pregnancy was greater than the physical sequelae endured.

## 4 Conclusion

AEP remains a rare diagnosis with significant maternal mortality from life‐threatening haemorrhage. Multidisciplinary working with peri‐operative imaging is key to prompt diagnosis and reducing the risk of maternal death. Ultrasound provision is globally imperative to site a pregnancy, ideally in the first trimester where management of EP is less surgically complex. POCUS with handheld devices may make this more achievable.

## Author Contributions

**Nina Cooper:** conceptualization, writing – original draft, methodology, investigation, writing – review and editing. **Alex Novak:** writing – original draft. **Georgina Gould:** writing – original draft. **Shaun Haran:** writing – original draft. **Maeve Tuomey:** data curation. **Maya Al‐Memar:** data curation, writing – review and editing, supervision. **Catriona Stalder:** data curation. **Tom Bourne:** writing – review and editing, supervision. **Joseph Yazbek:** writing – review and editing, supervision, investigation, data curation, conceptualization.

## Conflicts of Interest

GE Healthcare loaned the Vscan Air device to the department via author T.B.; however, we no longer have access to the device. GE Healthcare had no involvement in the study design, data collection, analysis, manuscript preparation, or decision to publish this report. The authors declare no financial or commercial conflicts of interest related to this work.

## References

[1] A. McDougall , A. Morin , T. Kuzmich , and F. Odejinmi , “Advanced Abdominal Pregnancy: Challenges, Update and Review of Current Management,” Obstetrician & Gynaecologist 24, no. 3 (2022): 195–204.

[2] R. George , E. Powers , and R. Gunby , Abdominal Ectopic Pregnancy Baylor University Medical Center Proceedings (Taylor & Francis, 2021). 10.1080/08998280.2021.1884932 PMC8224208 34219949

[3] A. Poole , D. Haas , and E. F. Magann , “Early Abdominal Ectopic Pregnancies: A Systematic Review of the Literature,” Gynecologic and Obstetric Investigation 74, no. 4 (2012): 249–260. 23108297 10.1159/000342997

[4] H. K. Atrash , A. Friede , and C. J. Hogue , “Ectopic Pregnancy Mortality in the United States, 1970–1983,” Obstetrics & Gynecology 70, no. 6 (1987): 817–822. 3684113

[5] A. Sayasneh , J. Preisler , A. Smith , et al., “Do Pocket‐Sized Ultrasound Machines Have the Potential to Be Used as a Tool to Triage Patients in Obstetrics and Gynecology?,” Ultrasound in Obstetrics & Gynecology 40, no. 2 (2012): 145–150. 22605511 10.1002/uog.11184

[6] M. Skendi , R. Liard , C. Besacier , et al., “Intrauterine Pregnancy Detection and Gestational Age Assessment During Early Pregnancy by a Handheld Point‐of‐Care Ultrasound Device Compared to a High‐End Ultrasound System. An Accuracy and Reliability Study,” POCUS Journal 7, no. 2 (2022): 225–231, 10.24908/pocus.v7i2.15458. 36896381 PMC9983726

[7] G. Mariani , J. Kasznia‐Brown , D. Paez , et al., “Improving Women's Health in Low‐Income and Middle‐Income Countries. Part II: The Needs of Diagnostic Imaging,” Nuclear Medicine Communications 38, no. 12 (2017): 1024–1028, 10.1097/MNM.0000000000000752. 28953209 PMC5704652

[8] G. Szabó and J. Rigó Jr , “Prenatal Ultrasound Diagnosis of Abdominal Pregnancy of Ovarian Origin,” Clinical and Experimental Obstetrics & Gynecology 46, no. 6 (2019): 977–979, 10.12891/ceog5000.2019.

[9] K. E. Elwell , J. L. Sailors , P. K. Denson , B. Hoffman , and C. Y. Wai , “Unruptured Second‐Trimester Ovarian Pregnancy,” Journal of Obstetrics and Gynaecology Research 41, no. 9 (2015): 1483–1486. 26017365 10.1111/jog.12726

[10] L. Muehlparzer , W. Arzt , T. Ebner , and G. Tews , “Secondary Abdominal Pregnancy With Live Birth,” Acta Obstetricia et Gynecologica Scandinavica 90, no. 3 (2011): 288. 21306308 10.1111/j.1600-0412.2010.01041.x

[11] Organization WH , Baseline Country Survey on Medical Devices 2010 (World Health Organization, 2011).

[12] L. Noguchi , M. Bucagu , and Ö. Tunçalp , “Strengthening Antenatal Care Services for All: Implementing Imaging Ultrasound Before 24 Weeks of Pregnancy,” BMJ Global Health 8, no. 5 (2023): e011170. 10.1136/bmjgh-2022-011170 PMC10255018 37257940

[13] S. Ngwenya , “Challenges in the Surgical Management of Ectopic Pregnancy in a Low‐Resource Setting: Mpilo Central Hospital, Bulawayo, Zimbabwe,” Tropical Doctor 47, no. 4 (2017): 316–320, 10.1177/0049475517700810. 28345398

[14] D. N. Nunyalulendho and E. M. Einterz , “Advanced Abdominal Pregnancy: Case Report and Review of 163 Cases Reported Since 1946,” Rural and Remote Health 8, no. 4 (2008): 1–8. 19053177

[15] G. Masselli , M. Derme , M. G. Piccioni , et al., “To Evaluate the Feasibility of Magnetic Resonance Imaging in Predicting Unusual Site Ectopic Pregnancy: A Retrospective Cohort Study,” European Radiology 28 (2018): 2444–2454. 29349699 10.1007/s00330-017-5237-6

[16] X. Zhang and C. Wang , “Predictive Value of PAPP‐A for Ectopic Pregnancy and Analysis of Related Factors,” Experimental and Therapeutic Medicine 22, no. 2 (2021): 1–7. 10.3892/etm.2021.10233 PMC8170667 34093757

[17] J. Farren , M. Jalmbrant , L. Ameye , et al., “Post‐Traumatic Stress, Anxiety and Depression Following Miscarriage or Ectopic Pregnancy: A Prospective Cohort Study,” BMJ Open 6, no. 11 (2016): e011864. 10.1136/bmjopen-2016-011864 PMC5129128 27807081

